# Liposomal Nanomedicine: Applications for Drug Delivery in Cancer Therapy

**DOI:** 10.1186/s11671-021-03553-8

**Published:** 2021-05-25

**Authors:** Foad Rommasi, Neda Esfandiari

**Affiliations:** Faculty of Life Sciences and Biotechnology, Shahid Beheshti University, Tehran, Iran

**Keywords:** Cancer treatment, Drug delivery, Therapeutic nanoparticles, Liposomal drugs, Nanomedicine

## Abstract

The increasing prevalence of cancer, a disease in which rapid and uncontrollable cell growth causes complication and tissue dysfunction, is one of the serious and tense concerns of scientists and physicians. Nowadays, cancer diagnosis and especially its effective treatment have been considered as one of the biggest challenges in health and medicine in the last century. Despite significant advances in drug discovery and delivery, their many adverse effects and inadequate specificity and sensitivity, which usually cause damage to healthy tissues and organs, have been great barriers in using them. Limitation in the duration and amount of these therapeutic agents’ administration is also challenging. On the other hand, the incidence of tumor cells that are resistant to typical methods of cancer treatment, such as chemotherapy and radiotherapy, highlights the intense need for innovation, improvement, and development in antitumor drug properties. Liposomes have been suggested as a suitable candidate for drug delivery and cancer treatment in nanomedicine due to their ability to store drugs with different physical and chemical characteristics. Moreover, the high flexibility and potential of liposome structure for chemical modification by conjugating various polymers, ligands, and molecules is a significant pro for liposomes not only to enhance their pharmacological merits but also to improve the effectiveness of anticancer drugs. Liposomes can increase the sensitivity, specificity, and durability of these anti-malignant cell agents in the body and provide remarkable benefits to be applied in nanomedicines. We reviewed the discovery and development of liposomes focusing on their clinical applications to treat diverse sorts of cancers and diseases. How the properties of liposomal drugs can be improved and their opportunity and challenges for cancer therapy were also considered and discussed.

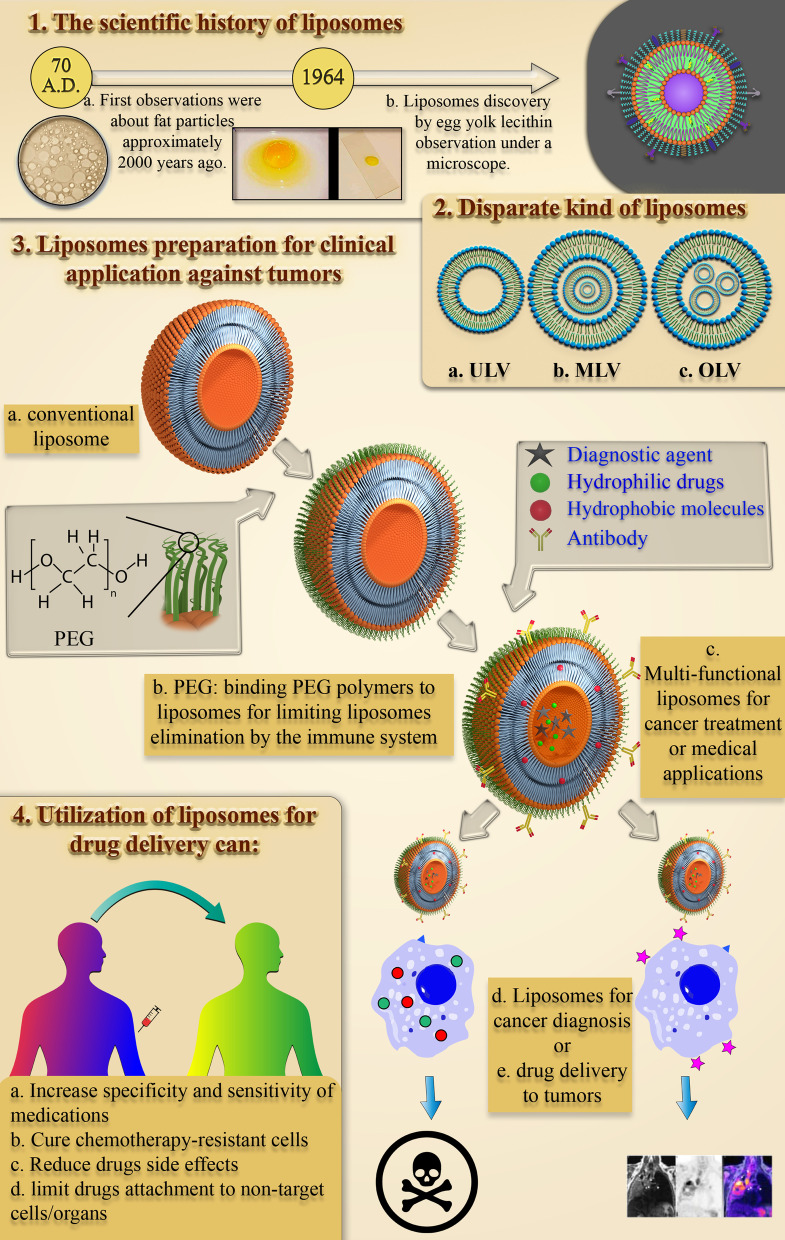

## Introduction

Cancer, a disease in which the healthy cells of body get out of normal condition and divide uncontrollably, is recognized as a big medical challenge in the current century. This complication is caused by the accumulation of environmental carcinogens or genetic mutations [1] and is recognized as a big medical challenge in the current century. Millions of people die every year due to cancer, and the number of new patients and the mortality rate is continuously growing [2]. According to the World Health Organization (WHO) reports, cancer was the second leading cause of mortality in 2018, and an estimation showed that about 9.6 million people died of various cancers in that year. In 2018, 1 in 6 deaths were approximately caused by cancer. About 70% of cancer deaths occur in developing and low-income countries. However, the incidence and fatality rate of cancer in developed countries should also be considered [3].

Chemotherapy with antitumor agents is known to be an important treatment for cancer [4]. Chemotherapy with free drugs is limited owing to a lacking of appropriate sensitivity and specificity. As a result, this limitation has prevented accurate treatment due to side effects and inhibited enough antitumor effect exertion [5]. Chemoimmunotherapy, a concomitant-combined treatment, has also been suggested as an effective and promising method for cancer therapy, explicitly treating tumor cells that are resistant to conventional medications. In recent years, a variety of conventional and advanced treatments have been discovered and applied to treat cancer. As an instance, to reduce the side effects of conventional anticancer drugs, specifically chemotherapy agents, various nanomedicines [6], including viral nanoparticles (VNPs) [7, 8], quantum dots [9], polymer nanomaterials [10], and liposomes [11] have been applied.

Among different nanomedicines, liposomes as spherical nanoparticles (NPs) have a particular structure. The presence of two aqueous and organic phases in the liposome constituent allows the entrapment of both kinds of hydrophilic and hydrophobic agents and creates a remarkable advantage for the liposome over many nanocarriers. One of the ways to enhance the specificity, bioavailability, and biocompatibility properties of antitumor drugs is to entrap them into diverse kinds of liposomes [5]. Over the past two decades, significant endeavors have been made to exploit liposomes for therapeutic purposes. Some of these drugs, such as DaunoXome® and Caelyx®, have been approved for general and clinical applications, while others are in the final production and approval stages [12].

Generally, there are different kinds of therapeutic liposomes such asImmunoliposomes and pH-sensitive liposomes. Immunoliposomes are a large group of nanomedical devices, also known as targeted drug delivery systems (DDSs), which have shown significant anti-malignant effects in studies and researches [13]. pH-sensitive liposomes are also known as a group of polymorphic liposomes, in which the structure and constituent molecules are altered by pH change, leading to the release of their drug content [14]. Moreover, liposomes, like other nanomedicines, have the potential to be utilized for tissue repair and regeneration, imaging, and diagnosis, in addition to drug delivery systems. The usage of liposomes in various aspects simplifies the identification, management, and treatment of diseases and cancers [15].

In this article, a summary of the findings on the discovery and structure of liposomes, various properties of liposomes, and liposomal drugs for cancer treatment on the market and their development are rendered. Ultimately, a report on the opportunities and challenges of liposomal nanomedicines utilization will be concluded, which can be highlighted as crucial issues to be noticed in scientists' future research, leading to the removal of the limitations and strengthening the positive points.

## Main Text

### The Scientific History of Liposomes: Discovery and Defining

**Fig. 1:**
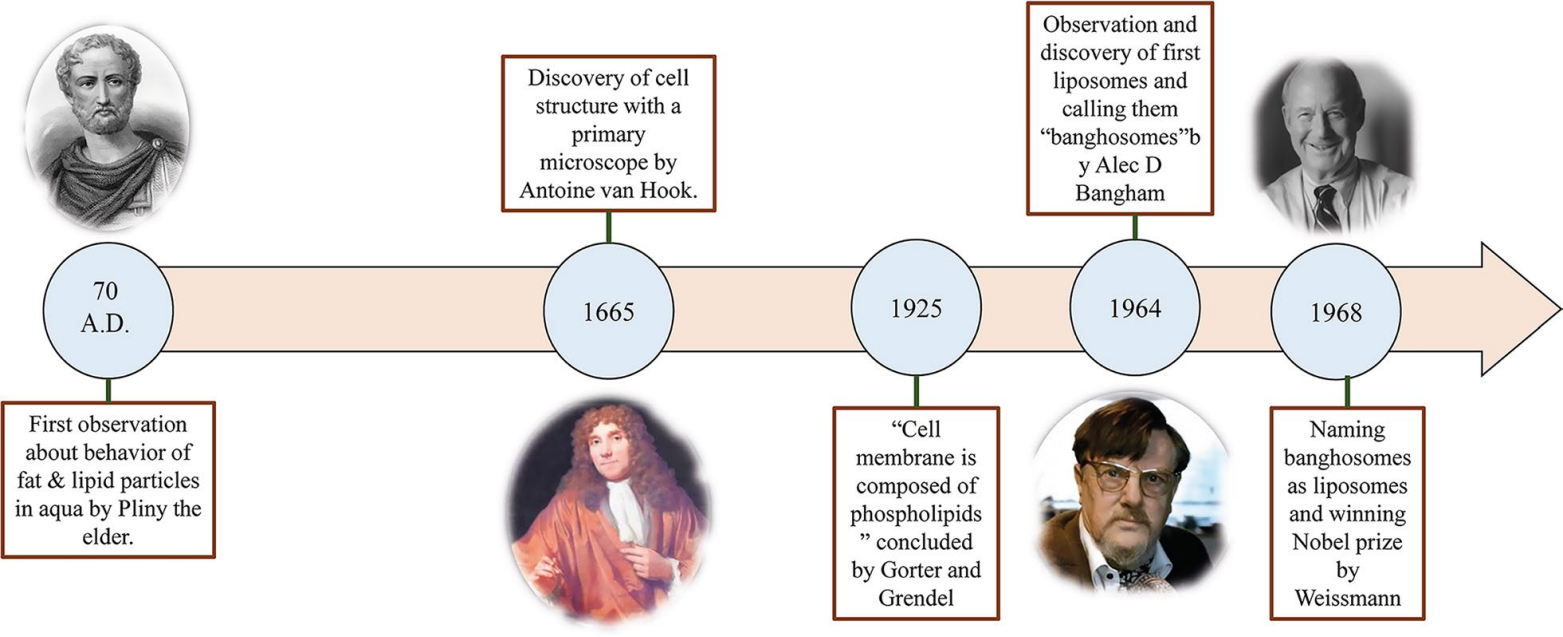
Diagram of observations that led to the discovery of liposomes. The historical and scientific trends of studying behaviors of lipid and fat particles in water and the observations leading to the liposomes discovery, along with images of the scientists involved in the event, Pliny the Elder [23], Anthonie Van Hook [24], Alec D. Bangham [25], and Gerald Weissmann [26], respectively, from left to right

It took about 1950 years from early studies on the structure and behavior of small lipid particles in an aqueous environment to the first US FDA-approved lipid-based drug delivery nanoparticle. The process of studying lipid and fat particle behavior in aqua began with the first observations by Pliny the Elder almost 2000 years ago [16]. In the late seventeenth century, the discovery of the cell by Anthonie Van Hook raised many questions about the structure of cells [17]. Then, Gorter and Grendel discovered the presence of phospholipid bilayers in cell membranes [18]. Subsequently, Singer and Nicolson later described the bilayer mosaic membrane model to explain the behavior of cell membrane phospholipids [19]. These scientific observations and hypotheses attracted the other scientists’ attention to fat-derived NPs. In the 1960s, Alec D. Bangham, who studied the effect of lipids, especially phospholipids, on the blood clotting process at the Babraham Institute [20, 21], observed accidentally the first liposomes and was surprised to see spontaneous spherical particles forming in the water [22]. Afterward, Gerald Weissmann, a visitor of Alec Bangham's laboratory who was aware of the results of Bangham's research, called the Smectic Mesophase observed by Alec "liposomes" instead of "banghosomes" and was awarded the Nobel Prize [22]. The scientific history of liposome discovery is summarized in Fig. 1.

### What Structures are Known as Liposomal Nanoparticles Nowadays?

There is an intense endeavor to define liposomal NPs and discover their properties reasonably. Nowadays, liposomes are defined as spontaneously-forming and spherical fragments which consist of a lipid bilayer membrane and a hydrophilic core.

Liposomes vary in size ranged from around 10 nm to 2500 nm (or 2.5 µm) [15]. However, most liposomes administrated for drug delivery are typically about 50 to 450 nm in size. Definitely, liposomes with much larger dimensions can also be utilized for medical applications [27]. Furthermore, liposomes are mainly composed of phospholipids. Phospholipids are a type of lipids, which are interestingly similar to triglycerides. In the structure of phospholipids, there is a hydrophilic pole and two hydrophobic chains. Thus, phospholipids are considered amphiphilic molecules.

**Fig. 2:**
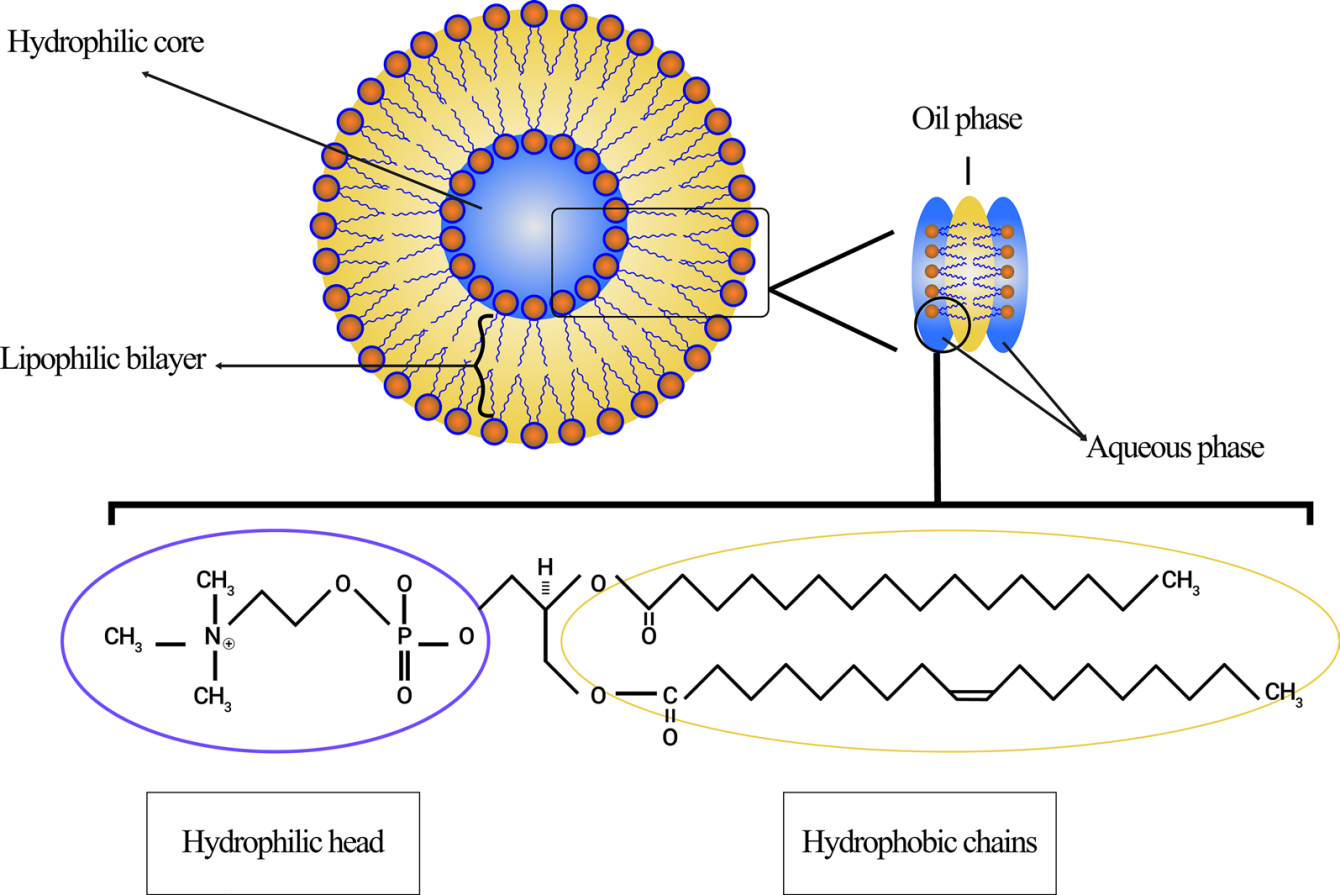
Schematic figure of liposomes originated from lecithin. Different regions of liposomes, including hydrophilic core and hydrophobic bilayer, are demonstrated. The structure of the lecithin molecule, its hydrophilic pole, and hydrophobic chains are specified

The liposome membrane of phospholipids mostly includes phosphatidylcholine (PC), sphingomyelin (SM), phosphatidylserine (PS), and phosphatidylethanolamine (PE), which are amphiphilic and have a strong tendency to form particular structures in water [28]. The physical reason for this phenomenon is the co-presence of a hydrophilic head (phosphate molecule) and two hydrophobic tails (fatty acids) in phospholipids. The phosphate group interacts with H_2_O polar molecules, while the hydrophobic tails escape from water molecules and interact with each other [29]. In this case, the non-polar chains are placed opposite to each other and make a bilayer, creating a lipophilic space between them. Accordingly, this lipophilic part structure of the liposomes can be applied to store hydrophobic agents and materials. Moreover, the hydrophilic section of the phospholipid is then directed toward the water molecules via molecular forces, like Hydrogen bonds, Van der Waals, etc., which appear between them. These lead to form a hydrophilic area inside the liposomes. The structure of the lecithin molecule, as a natural phospholipid that is abundant in egg yolk and able to form liposomes in water and various regions of a liposome, is shown in Fig. 2.

As well, the spherical structure of liposomes after dissolution in water, depending on the type of molecules, temperature of the aqueous medium, molar concentration, and the presence of other substances, such as ions, determines its ultimate shape [30]. The predominant physical and chemical attributes of a liposome are the net properties of its composing lipids, especially phospholipids and the other molecules that make it up. These properties include permeability, surface charge density, and overall size [31].

### Disparate Types of Liposomes Classification

Since liposome discovery, these structures have always been utilized as an essential part of biological, biophysical, biochemical, or pharmaceutical researches.

**Fig. 3:**
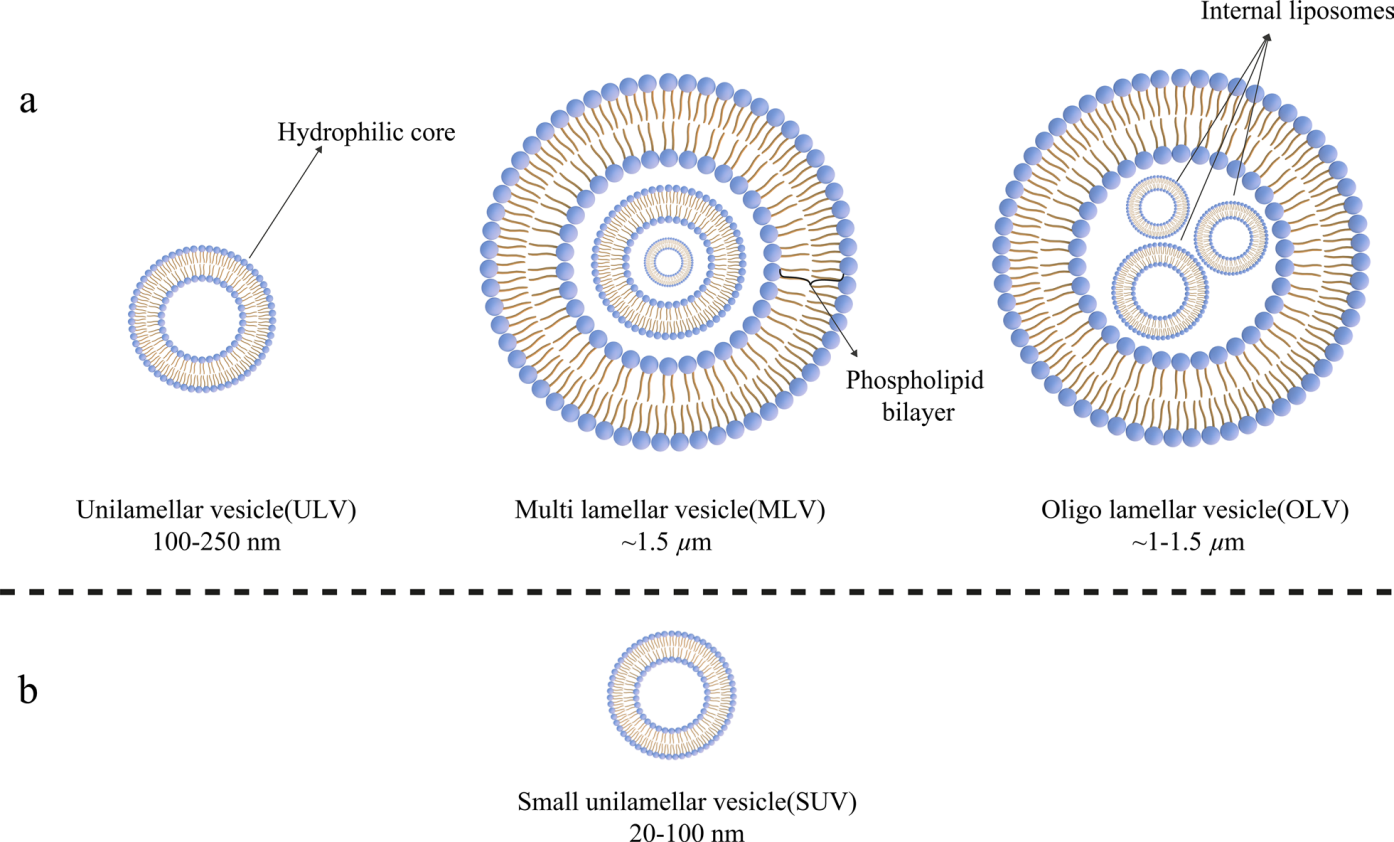
Classification of liposomes according to various criteria: **a** Liposomes are divided into three categories in terms of size; **b** The small unilamellar vesicle (SUV) structure, as a member of unilamellar vesicles (ULVs), which has a noticeable small size

Today, liposomes can be categorized based on their size, the number of their phospholipid bilayer, synthesis procedure, and preparation mechanism. In terms of size, liposomes can be divided into three groups: small, medium, and large. Considering the number of membrane layers, they can be unilamellar vesicles (ULVs), oligolamellar vesicles (OLVs), and multilamellar vesicles (MLVs). In this regard, ULVs are liposomes composed of one phospholipid bilayer measuring about 50 to 250 nm, while MLVs are much larger, about 0.5–1.5 µm, and include several phospholipid bilayer membranes [32]. Different methods of synthesis cause margins between these two groups. In terms of application, ULVs also have a large hydrophilic environment internally, which makes them suitable for the entrapment of hydrophilic drugs. Small unilamellar vesicles (SUVs), like ULVs, are composed of one phospholipid bilayer, but in terms of dimension, they are less than 100 nm in size [33, 34]. From a morphological point of view, OLVs are liposomes that are composed of two to five vesicles that may have identical or different sizes. In the structure of OLVs, the vesicles are all enclosed in one large phospholipid bilayer without being inside each other. OLVs usually are about 0.1–1 µm [33–35]. In contrast to ULVs, MLVs are not ideal for the delivery of hydrophilic substances. MLVs are mostly exploited for the delivery of hydrophobic agents [36]. Different types of liposomes are illustrated in Fig. 3.

### Liposomes Preparation Methods and Development of Their New Generations

In contrast to gold nanoparticles as hard NPs, liposomes are soft NPs [37] and can be synthesized through different methods. For example, MLVs and ULVs have distinct mechanisms of preparation. In most of these methods, a particular solvent (such as chloroform or methanol, etc.) is used to solve lipids intended to form liposome membranes (with the desired molar ratio) in a round-bottom flask (RBF). For instance, hand-shaking is the primary procedure to synthesize MLVs [38]. During this procedure, which is also recognized as lipid film hydration, lipids are added to an organic solvent. Then, the solvent is evaporated by a rotary device, and the solid product is lipholyzed. Ultimately, liposomes are synthesized following hydration and extrusion methods [39]. Other methods of liposome synthesis include sonication, reverse phase evaporation, French pressure cell, freeze-drying and, membrane extrusion [38, 40].

Furthermore, liposomes can also be ordered in different categories based on their discovery and development over time. First-generation liposomes are generally named conventional or classical liposomes. Problems, which were observed when using conventional liposomes as therapeutic NPs, were identified so quickly in vivo. One early examined issue was the limitation of drug entrapment into liposomes. In other words, many drugs were not capable of being stored inside first-generation liposomes [41]. Along with the great desire to survey the structure and properties of liposomes, such as stability, therapeutic efficacy and, the possibility of clinical applications, these challenges led to the development of second-generation liposomes by altering constituent lipids, surface charge, net weight, and total volume [42]. To be precise, second-generation liposomes are mainly synthesized by adding some hydrophilic polymers to conventional liposomes for the enhancement of their shelf-life in body fluids to make them appropriate candidates for drug delivery systems. This kind of liposome can be divided into two groups: non-specific long-circulating liposomes or ligand-targeted long-circulating liposomes [43].

Archaeosomes, as a novel generation of liposomes, are made up of the archaeal membrane lipid and synthetic phospholipids analogs. In the last decade, extensive and considerable efforts have been made to investigate the potential of archaeosomes to be employed in drug and vaccine delivery. The structural nuclei of archaeal-type lipids are di-ether or tetra-ether molecules with saturated, phytanyl chains which contain about 20 to 40 carbons. These carbon chains attach to the ether bonds of sn-2,3 carbons of the backbone glycerol found in archaeol or caldarchaeol. As mentioned above, these particles can also be immensely used in drug delivery for neoplastic complications, allergies, and infections, as well as vaccinations [44].

### Evaluation of the Biomaterial Characteristics and Physicochemical Properties of Liposomes

As mentioned earlier, despite the widespread advances in medical sciences, the treatment of some diseases, especially cancer, still faces formidable challenges due to the inefficient therapeutic agents and methods. Adjusting the injected drug dose to influence tumors is a tense issue due to the narrow therapeutic window of anti-cancer agents. In other words, a little distance between the therapeutic and the toxic dose, as well as inappropriate sensitivity and specificity, have created a great demand for advanced remedial procedures [42].

**Fig. 4:**
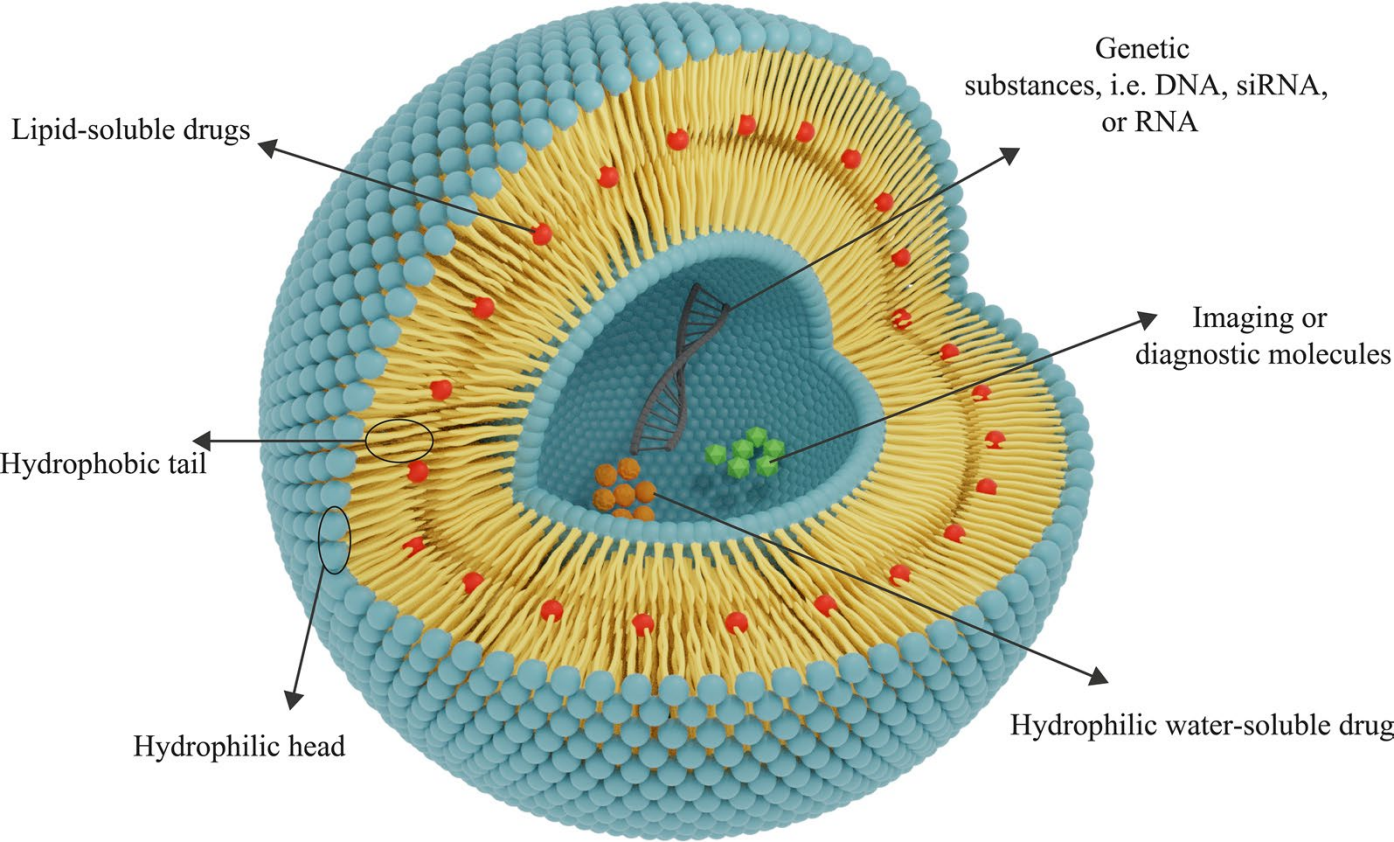
The general structure of the liposomes consists of phospholipid layers. Depending on the hydrophilicity–hydrophobicity of a drug, the appropriate kind of liposomes for its delivery will be determined. Hydrophilic drugs are entrapped in the central hydrophilic nucleus, and hydrophobic drugs are placed in the lipophilic area. Liposomes can also be utilized for the delivery of genes

Moreover, the utilization of nanomaterials for drug delivery to tissues has drawn attention recently. Biocompatibility and biodegradability are two essential features from biomaterial characteristics for the avail of nanomaterials in delivery systems. Biocompatibility is required to inhibit therapeutic NPs from damaging the body tissues and systems and, biodegradability is urgent to break down NPs into non-toxic compounds and remove them simply from the organs [15]. After liposomes detection, scientists began to apply them as nanomaterials for drug delivery. As it is mentioned, liposomes have two required biomaterial properties for therapeutic purposes: biocompatibility and biodegradability [36]. Also, liposomal NPs have other characteristics that make them suitable for this purpose. For instance, due to the specific structure of liposomes, both groups of hydrophilic (water-soluble) and hydrophobic (lipid-soluble) drugs can be encapsulated in them. Furthermore, the presence of a phospholipid bilayer membrane in liposomes protects the agents stored in liposomes from various phenomena and damages, such as enzyme degradation, biological inactivation by immunological structures, and chemical changes in vivo. This point has two significant pros: first, the structure of the molecules entrapped in the liposome is preserved before reaching the target tissue, and no modifications are made into it and, second, other healthy and non-target tissues are protected from exposure to the drug due to the liposome membrane and, cannot be influenced by these agents [42]. Liposomes can also be applied for delivering genetic materials, such as DNA, RNA, etc. and, gene therapy purposes. Liposomes used for this aim can be composed of cationic, anionic, neutral lipids and, phospholipids or a mixture of them [45]. Some diagnostic and imaging agents, such as carbon dots, can be utilized for cancer detection and imaging in the combination of liposomes or singly [46]. Although carbon dots are partially approved for clinical application and exploited in investigations, cytotoxicity remains a challenging barrier for their wide application [47]. The general structure of liposomal drug NPs is shown in Fig. 4.

The membrane-forming phospholipids present in liposomes are non-toxic compounds and can be synthesized in an extensive range of sizes. The physicochemical attributes of liposomes are dependent on their constituents. Thus, liposomes with the desired properties can be synthesized by adding certain compounds, such as cholesterol, polyethylene glycol (PEG), etc. Besides, the membrane of liposomes is impermeable to giant molecules, which helps to retain material within the liposome better [48]. All the mentioned features of the liposome introduce it as an appropriate nanomaterial for exploitation in the delivery of therapeutic agents for treating various diseases, especially cancer.

Gregory Gregoriadis, as one of the pioneer researchers in this field, proposed the hypothesis of using liposomes for the drug delivery systems and stated that drug compounds could be entrapped in liposomes [49]. The appropriate biomaterial and physiochemical properties of liposomes have been reported. As an instance, a survey on liposomes containing the antitumor drug cytosine arabinoside used in animal models has shown a significant increase in mice's lifetime with L1210 leukemia [50]. By applying liposomes, a sufficient dose of the active form of the drug can be delivered to the target site in a protected manner [42].

### Enhancement of Specificity and Sensitivity of Liposomal NPs for Therapeutic Usages

As it is mentioned earlier, using various molecules and polymers makes it possible to change in the structure and membrane of liposomes, and through this, new functionalities can be added to liposomes or their properties can be modified [51]. Both prolonging the circulation of liposomes in the blood and increasing their ability to accumulate in specific tumor tissue or pathological site via the EPR effect are the first important features that should be taken into account because of the high rate of clearance of liposomes. The conjugation of PEG molecules to liposome membranes through chemical conjugation has been used sequentially to add this functionality to liposomes [52]. The importance and role of ethylene glycol polymers in increasing the half-life of liposomes, especially the liposomal therapeutic NPs in body fluids, such as blood, were expressed about 20 years ago [53].

**Fig. 5:**
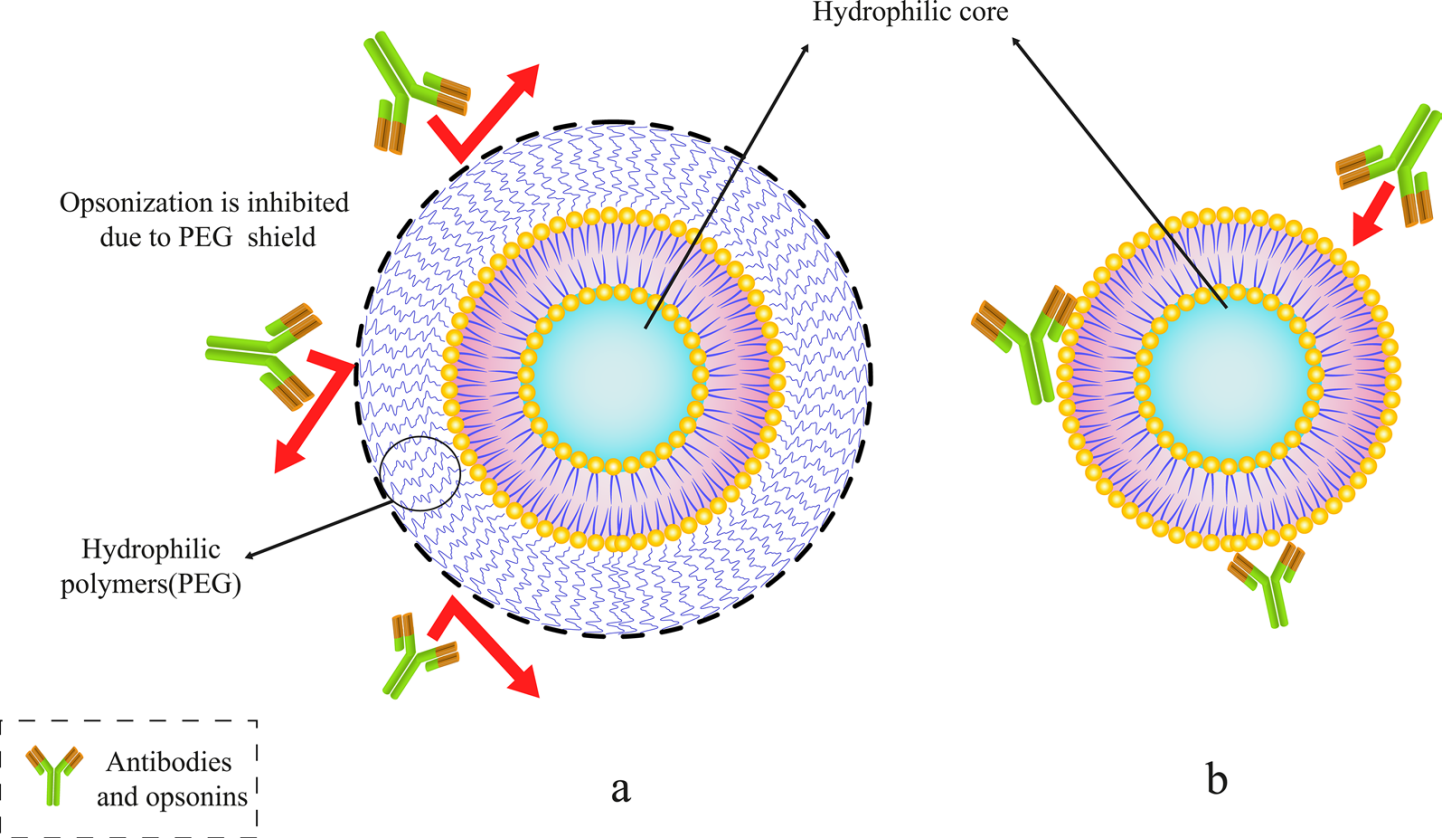
Conjugating a specific polymer such as polyethylene glycol (PEG) to liposomes. **a** PEGylated liposomes with PEG polymer molecules shield. **b** Conventional liposome trapped by antibodies and opsonins

Abuchowski and McCoy made the first attempts to extend the half-life of liposomes in the bloodstream by conjugating PEG to their structure. As a result, their efforts generally increased the circulation time of liposomes and their half-life in the bloodstream [54]. After a few months, other researchers investigated the possibility of reducing the high speed of clearance of liposomes by mononuclear phagocytosis system cells (MPS). By attaching PEG to the surficial molecules of the liposomes [53], it is expected that the liposomes circulation in blood improves. There is a multitude of articles in this field. Moreover, unlike conventional liposomes, PEG-coated liposomes demonstrated dose-independent pharmacodynamics properties [55]. Among various polymers, PEG molecules are one of the polymers that can be attached to the liposome surface to prolong their shelf-life in vivo. Other polymers can also be used for this purpose [56]. Figure 5 demonstrates how the PEG polymer molecules can protect the liposome from antibodies and also prolong its life in the bloodstream.

As mentioned, it is noteworthy that other molecules can be exploited to prolong the circulation of liposomes in addition to PEG. Furthermore, polyoxazolines polymers are among the substances used to modify the liposomes membrane to enhance their half-life. In this regard, Woodle et al. were the first group to apply poly[2-ethyl-2-oxazoline] (PEOZ) to synthesize stealth liposomes. Their results testified a reduction in the elimination and uptake of poly[2-ethyl-2-oxazolylated] PETOXylated-liposomes injected into rats by hepatic-splenic cells [57]. Their outcomes indicated that the conjugation of other polymers such as poly[2-ethyl-2-oxazoline] and poly[2-mthyl-2-oxazoline] (PMOZ) could have PEG-like effects in increasing the half-life and prolonging the circulation of liposomes in vivo. They also compared the bio-distribution of PEG-, PEOZ-, and PMOZ-conjugated liposomes in various organs and systems. The consequences also demonstrated that the bio-distribution of all these liposomes in blood and spleen was almost the same, but in the liver, the distribution of PMOZ was much lower than the others [57].

Pain et al. bound dextran molecules to the surface of ULVs. Their results showed that dextran-conjugated liposomes, in comparison with conventional liposomes, had more extended circulation and lower absorption and uptake by the liver and spleen. This consequence testified that dextran molecules, in addition to prolonging the shelf-life of liposomes in the body, could also be applied for increasing the stability and regulate the rate of drug release from liposomes [58].

The second issue that should be noted is the fluidity and stability of liposomes. Other lipids, including cholesterol, can be used in the frame of liposomes. Cholesterol may occasionally be substituted for some compounds in the phospholipid bilayer to enhance some properties of the liposome. Nevertheless, it has been proven that modifying the content of the liposome bilayer and replacing some of the phospholipid molecules with certain compounds, especially cholesterol, can reduce the fluidity of liposomes [59]. Besides, the presence of cholesterol in the membrane of liposomes increases the stability of their structure (both in vivo and in vitro experiments). It also reduces the permeability and the possibility of leakage of entrapped substances. Cholesterol is a hydrophobic steroid that interacts with the hydrophobic chains between phospholipid bilayers to stabilize its structure when present in the membrane of liposomes. This action of the cholesterol is substantial when exploiting liposomes clinically in vivo because it prevents the liposome from converting to high-density lipoprotein (HDL) and low-density lipoprotein (LDL) in the body. Moreover, lipid structures present in the blood and intracellular fluids can have an impression on liposomes. Lipoproteins such as LDL and HDL affect injected liposomes and cause lipid transfer and rearrangement of their membranes. They also drastically reduce the stability of drug-containing liposomal NPs [12]. It is noticeable that other materials, such as DNA and other molecules utilized in liposome membrane for therapeutic applications, have to be anchored to the cholesterol in the membrane. Adding various substances to liposome membranes is one way to create positive features in liposomes [42].

The third essential features that should be considered are the sensitivity and specificity of liposomes for accurate identification and specific binding to target cells. By binding compounds, like monoclonal antibodies, F_ab_ fragments and other conjugative molecules such as transferrin and folate, it is possible to enhance the specificity of liposomes, resulting in specific binding to tumor cells [60]. In addition, enhancement of the specificity and sensitivity of drug nanocarriers, specifically liposomes, has been investigated before. For instance, Mohammad J. Akbar et al. studied peptide-PEG-lipid-conjugated liposomes to treat small cell lung cancer (SCLC). Their results showed that binding the Gastrin Releasing Peptide Receptor (GRPR) antagonist peptide to liposomes could increase the specificity and accumulation of these liposomes in GRPR-expressing cells. They also claimed that these liposomes attached to peptides could be applied to treat lung cancer cells due to the upregulation of GRPR-expressing genes in them [61].

Eventually, medications and drugs were added to PEGylated liposomes because of their appropriate properties, and now, these liposomal structures have been established for industrial-clinical utilization [62]. Antibodies were also used in early studies to increase liposomes ability to bind to target cells [63]. In this case, receptor-mediated endocytosis was performed by liposomes to enter the cell [64]. Meanwhile, various methods have been developed to bind antibodies to liposomes [65]. Studies on the antibody-conjugated liposomes have proven that the toxicity of anticancer drugs against cultured tumor cells increases with the conjugating of antibodies to liposomes surfaces [66]. When antibodies were applied on the surface of PEG liposomes, the relic of antibodies to attach to their target receptors was veiled by PEG polymers, especially when the side chains attached to the PEG were long [67]. Therefore, the simultaneous use of PEG and antibody for liposomes medication and its disadvantages should be considered by scientists.

The fourth important factor in the therapeutic application of liposomes is the etude of the release of drugs entrapped in them. Adjustment of liposomes for the extrication of drugs within them affected by the abnormal conditions of the damaged tissues is one of the crucial issues in administrating liposomes clinically. Furthermore, the usage of temperature-sensitive compounds, pH, or a specific metabolite in the surface of liposomes that can bind to the tissue and membrane surface of the target cells is a method to release these drugs precisely. Utilizing this method can result in the specific effect of liposomes on the membrane surface of target cells and also release the drug content inside them [68].

The releasing rapidity of compounds entrapped in liposomal NPs is the fifth substantial criterion for adjusting the dose of drugs available at the target site. One of the essential objects that should be considered for the proper usage of all kinds of drug delivery systems, including liposomes, is the releasing rate of drugs and regulation. With regard to liposomal drug delivery systems and NPs, it is worth mentioning that the encapsulated substances in the liposomes are not biologically available and can only be bioavailable while it is released from the initial state. Therefore, drug-containing liposomes can provide the ability to increase the concentration of bioavailable drugs for cancerous tissues and to improve the quality of treatment and therapeutic efficacy can be achieved on condition that the rate of drug release from the liposome is adjusted [69]. Furthermore, it has been proven that changing the liposome bilayer content and replacing some phospholipids with certain compounds, especially steroid molecules like cholesterol, can decrease the permeability and unintended leakage of the compounds stored in them [70]. Consequently, this advantage can be exploited to adjust the release rate of the encapsulated compound. Once released, the drugs must penetrate sufficiently into the cell and make the necessary physiological-biochemical changes to exert their impact.

**Fig. 6:**
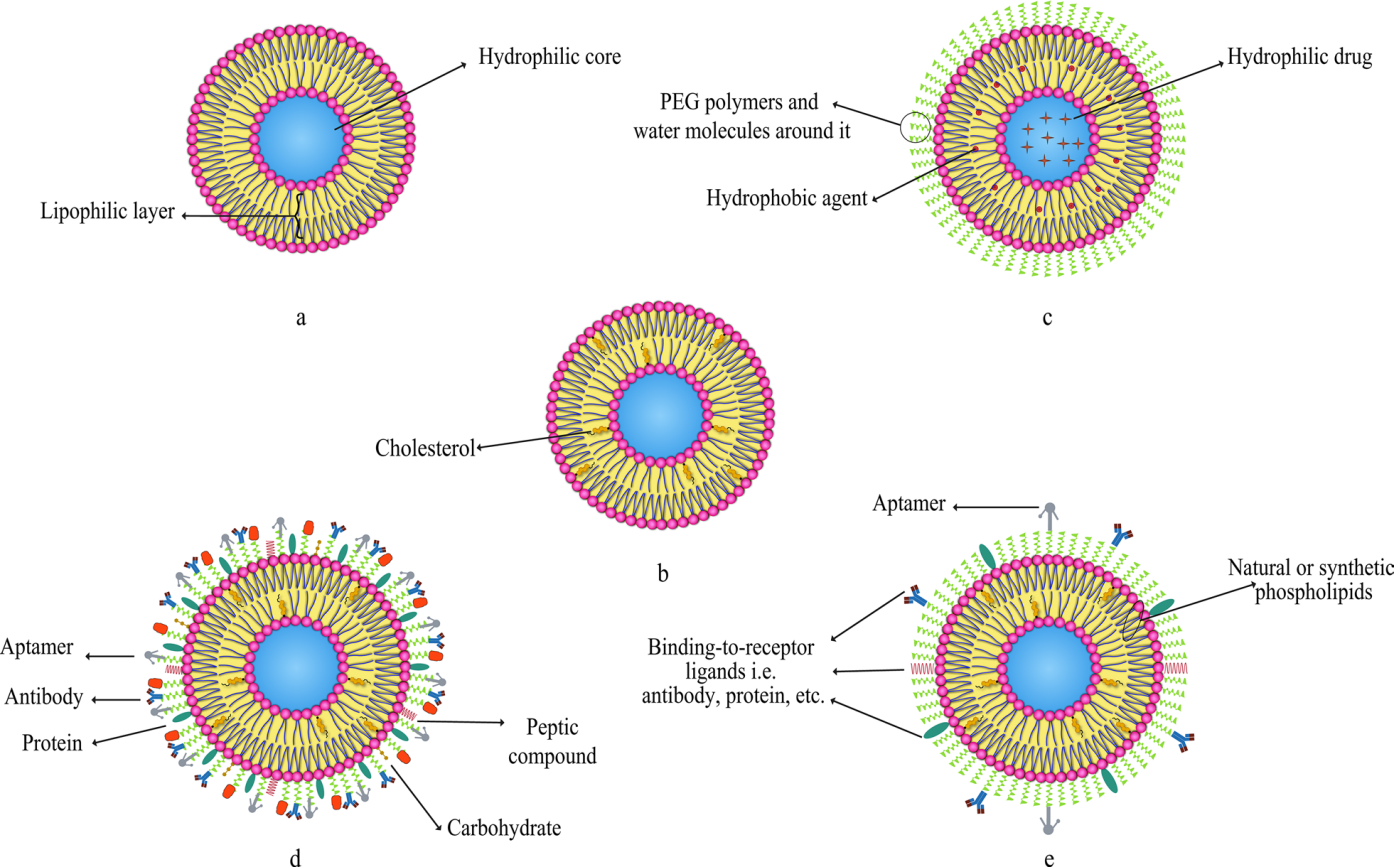
Various kinds of liposomes. **a** Conventional liposome; **b** cholesterol-conjugated liposome; **c** PEGylated or stealth liposome; **d** ligand-targeted liposome; **e** multi-functional liposome

As it is mentioned earlier, various compounds, including aptamers, can be conjugated to liposomes. In this regard, Mohammad Mashreghi et al. applied anti-epithelial cell adhesion molecule (anti-EpCAM) as an aptamer to functionalize Caelyx® liposomes. Their experiment outcomes determined that functionalization of Caelyx® with this aptamer could enhance the merits of this liposomal drug and made it a viable option for cancer treatment [71]. Figure 6 shows the structure of different types of liposomes that are used in vitro or for clinically scientific purposes schematically.

The passage of drugs through lysosomes to enter cells (which have low pH and many degrading enzymes) is the sixth most important factor for the practical application of conjugated medicines to liposomes. To protect therapeutic agents from unwanted conversions in extracellular and intracellular space, cell-penetrating peptides are attached to the liposome surface [72].

### On Liposomal Drugs Pharmacology: Pharmacokinetics and Pharmacodynamics

The assessment of pharmacological attributes, as an essential part of medicine and pharmaceutical science, is required not only to gain a better understanding of liposomes pros and cons as drug carriers but also to confirm and evaluate them in clinical trials. The pharmacological properties of liposomal drugs and their interactions with the body can be examined in two various aspects: pharmacokinetic (the effect of the body on therapeutic compounds) and pharmacodynamics (how medications act and impact the body and cellular pathways) [73]. In general, the utilization of liposomes for drug delivery in cancer treatment or other disorders requires the elevation of these agents' effectiveness on the one hand, and reducing their toxicity toward normal tissues on the other hand. Subjects such as the proper administration route of NP-based drugs, their circulation in the bloodstream and half-life, their biological distribution in tissues, and their cellular metabolism, as well as their elimination, metabolization and clearance, have been studied in the field of pharmacokinetics [74]. The pharmacokinetics of liposomes primarily study the bioavailability of liposome-conjugated drugs in various body fluids and tissues. Indeed, the study of chemical decomposition and biological excretion, and liposome uptake and purification are also considered in pharmacokinetics. The results of studies on pharmacological advantages of using liposomal drugs (regardless of the type of liposomes applied in the DDS) instead of free drugs showed that:

Primarily, liposome can modify the drug release profile to a sustained release, and consequently, reduce the requirement for constant injection. Secondly, it can extend the presence of the drug in the bloodstream and body fluids, and as a consequence, increase its half-life. Thirdly, it has the potential to lead to better bio-distribution in cancerous tissues while reducing drug influences on healthy tissues due to limited particle size to cross the Endothelium of healthy capillaries. Ultimately, it reduces drug metabolism and inactivation in plasma before reaching the target tissue, in addition to its positive effect on the clearance of drug metabolites [75, 76].

However, some changes are required in the pharmacokinetics of liposomes to increase their solubility, specificity, and sensitivity. These modifications enable them to overcome chemotherapy-resistant cells, enhance the efficacy and half-life. Moreover, their toxicity or unintended metabolic compounds product as a result of their metabolization should be decreased by these modifications [77].

After consuming liposomal drugs administration, they enter the body and circulate in the bloodstream with a specific half-life. Their size and formative composition determine the half-life of liposomal medications. Moreover, rapid clearance of liposomal drugs from the body can reduce their duration of action and therapeutic index. As aforesaid, appending hydrophilic polymers such as PEG to liposomes is able to decrease their clearance rate and solve this challenge [78]. Also, it is possible to adjust the fluidity and drug-release rate of the liposome membrane by adding cholesterol molecules.

The application of liposomes for drug delivery may lead to some changes in drug pharmacokinetics [79]. The ability of liposomes to change the pharmacokinetic properties of the various drugs and medications is one of their significant benefits in drug delivery systems [80]. Concerning the process of liposome clearance and elimination, it is obvious that liposomal structures are affected by plasma proteins after being administered. For instance, after injection of liposomal nanoparticles opsonins are adsorbed on the surface of the liposomes. Opsonins are plasma protiens which mostly include immunoglobulins and fibronectin [42]. Opsonins presence on the surface of liposomes will result in their elimination by MPS, as one of the significant elimination section of various drugs from blood and body fluids. They also clear liposomes through the attachment of some receptors such as complement C3b and Fc to opsonins-liposomes complex [81]. Various tissues and cells such as liver kupffer cells, macrophages present in the spleen, bone marrow, and lymph nodes are involved in the clearance of liposomal NPs [82].

According to the International Union of Pure and Applied Chemistry (IUPAC) definition, pharmacodynamics refers to the study of the pharmacological impact of compounds on living systems and the biochemical and physiological consequences of these effects [83]. The increased elusion to identify therapeutic agents when encapsulated in liposomes has been recognized as one of the pharmacodynamical benefits of liposomes utilization [84].

Furthermore, the physicochemical characteristics have significant influences on the pharmacology of liposomal drugs. The particle size, electrical charge of membrane, and the composition of membrane lipids are some of these physicochemical properties that can affect the pharmacokinetics and pharmacodynamics of the agents. Firstly, there is a direct relationship between the particle size of nanoparticles, including liposomes, and their clearance rate. By increasing the size of NPs, their elimination rate by the immune system and MPS cells will also enhance [85]. Secondly, it is worth mentioning that the net charge of liposome membranes is a consequence of the electrical charge of phospholipids and their other constituting particles that made them up. As a result, a rise in the membrane charge is associated with enhanced clearance rates of these agents [86]. The composition of membrane lipids and other structural features (such as hydrophilic core radius) also remarkably affect the pharmacokinetics of liposomal drugs [87].

More importantly, it has been hypothesized that different types of liposomes exhibit distinct drug kinetics/dynamics depending on their various structures. The drug release rate also rests on the number of phospholipid bilayers and the content of loaded drug compounds. It is also contingent upon the hydrodynamic diameter, total volume, and other pharmacokinetic properties as well [88].

### Administration Route of Liposomal Drugs

Like many different drugs, NP-based liposomal medicines can be administered from a wide variety of routes. In other words, oral consumption [89] and distinct injection methods such as intravenous (I.V.) administration and various local injections are among the common administration routes of liposomal drugs [90]. The usage of nanoparticles, including liposomes, for drug delivery via oral administration has been highlighted as an effective strategy since the nanoparticles increase the bioavailability of medicines, improve their interaction with cells, and prevent any modifications in the molecular structure of the drug due to enzymes and gastric juices in the gastrointestinal tract. Moreover, they have the ability not only to enhance the release of remedial molecules into the mucosal and epidermal layer but also to protect drugs from unwanted changes during the first pass effect [89]. Intravenous injection is used as the primary administration route for many liposomal drugs approved by the FDA or other authorities [42]. On the other hand, subcutaneous (S.C.), intradermal (I.D.), intraperitoneal (I.P.), and intramuscular (I.M.), classified under the title of the local injection, are also utilized for administration of liposomal drugs [90–92].

### Liposomal Drugs Fate In Vivo and Their Targeting Mechanism of Action

**Fig. 7:**
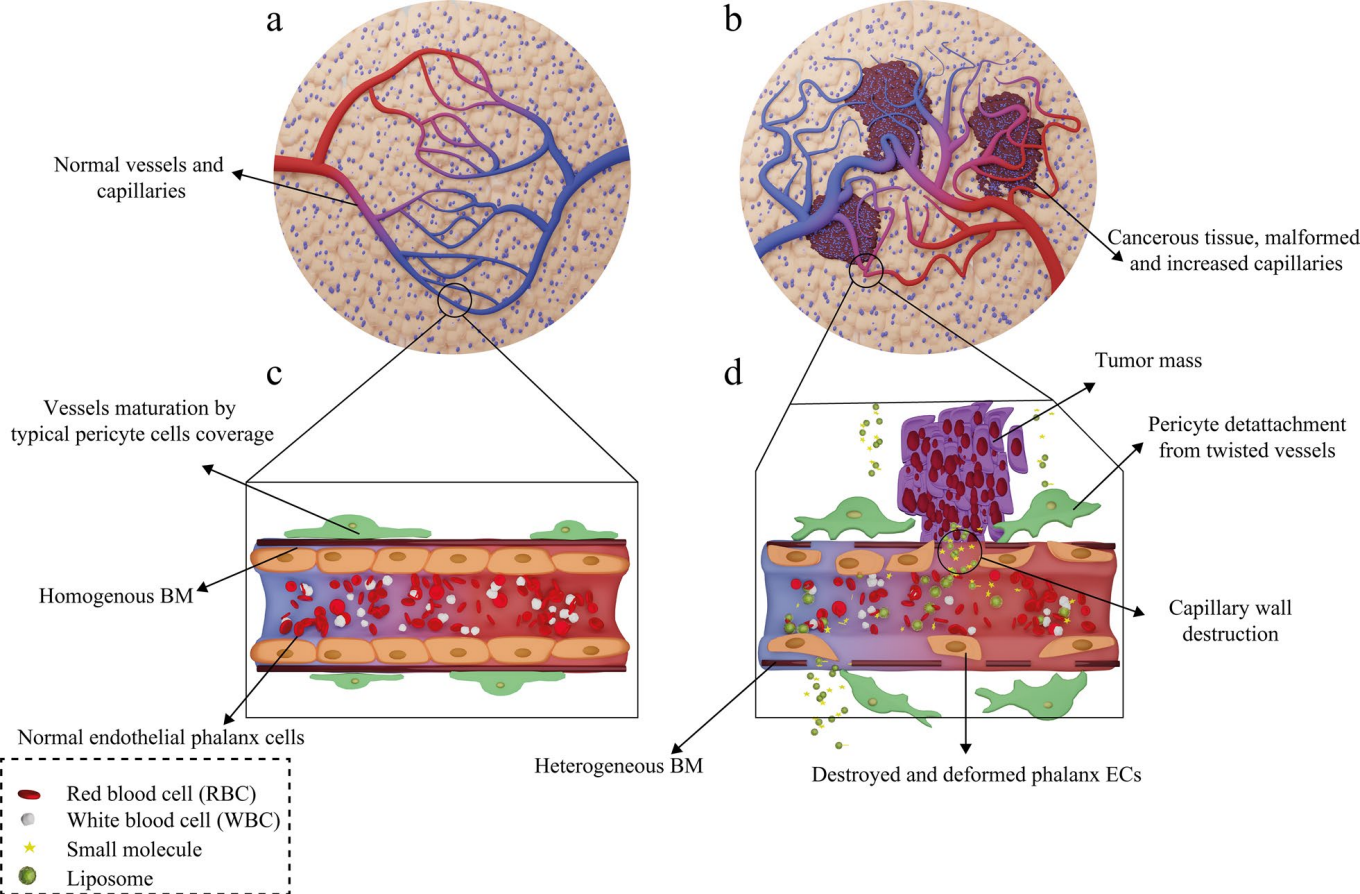
Mechanism of action of the drug-containing liposomes on tumor cells via EPR effect. **a** Healthy tissue and its normal capillaries; **b** cancerous tissue with increased-deformed vessels; **c** structure of normal and healthy vessel; **d** destructions and deformed capillary in tumor tissue

Following administration of the liposomal drugs, they reach the pathological lesions at the target site through the bloodstream and accumulate there. The mechanism of action of liposomal drugs on tumors starts with their accumulation at the target site, uptake of them by tumor cells, and the release of free drugs [93]. Subsequent to entering the body, liposomal drugs reach the tumors through various targeting mechanisms of action and then interact with cells in different ways [94]. In general, tumor-targeting mechanisms are divided into two categories: passive and active targeting. Passive targeting refers to the mechanisms in which liposomes are spontaneously accumulated at the tumor site and interact with target cells without the presence of a specific ligand [95]. The effect of enhanced permeability and retention (EPR) has been suggested as the most critical passive targeting mechanism. To be precise, the spontaneous accumulation of therapeutic NPs and liposomal drugs at the tumor site is called the EPR effect [96]. This phenomenon can be assigned to the leaky nature of tumor tissue vessels, unlike normal tissue capillaries, which makes them permeable to molecules and NPs. Consequently, This ultimately leads to the accumulation of drug compounds in these tissues and the effect of EPR [97]. The ultimate fate of the drug in the intracellular fluid and cytoplasm of tumor cells depends on several factors such as release mechanism, nanocarrier constituents, and molecule structure [98]. In healthy tissue, the number and the shape of capillaries are proportionate and normal, respectively. However, in cancerous organs, unlike healthy tissue, the number and the structure of capillaries are higher and deformed, respectively, because of the angiogenesis process. Moreover, the tumor capillaries structure is destroyed, and the endothelial phalanx cells are diminished. As a result, the volume of plasma fluid leaking into the intercellular space will be enhanced. In healthy tissue, however, capillary phalanx cells retain cellular tight adhesions, preventing NPs, small molecules, and liposomal drugs from seeping into the intercellular space [99]. The EPR effect in cancerous capillaries and their difference with normal and healthy tissue vessels are illustrated in Fig. 7.

On the other hand, active targeting has attracted considerable attention as one of the targeting mechanisms of action owing to its appropriate effectiveness and high specificity. Active targeting includes various types and is also generally aimed to reduce the off-target impacts of liposomal NPs on healthy cells and non-target tissues [95]. In this method, molecules such as monoclonal antibodies, small molecules, signal peptides, vitamins, particular carbohydrates, glycolipids, or aptamers are generally utilized for surface modification of liposomes [100, 101]. Moreover, active targeting can be split into various subtypes according to diverse features. For instance, it can be classified into two general categories:

1. Targeting tumor cell and cancer tissue receptors: This method relies on conjugating specific molecules to the membrane surface of liposomes, making them able to bind to special or overexpressed receptors on cancer cells [102]. In cancer cells, upregulation of different genes causes an increase in the expression of specific cell surface receptors in response to enhanced metabolic demands for rapid cell proliferation [103]. In active targeting, particular molecular modifications can be applied for targeting specifically the overexpressed surface receptors of cancer cells, such as folate receptor (FR), transferrin receptor (TfR), or Epidermal growth factor receptor (EGFR) [95]. In this regard, the role of folate receptors in cancer cells is to increase folic acid uptake [104], whereas transferrin receptors bind to transferrin (as a free molecule with 80 kDa weight in serum) and cause endocytosis of this monomeric glycoprotein to occur [105]. Moreover, EGFR receptors are a class of tyrosine kinases involved in cellular processes such as tissue differentiation and repair. The expression of this receptor in cancer cells is significantly increased due to its involvement in processes such as angiogenesis, cell proliferation, and metastasis [106].
2. Utilizing tumor microenvironment as the target: In this method, changes in the surface of liposomes are exploited to enable them to target signal peptides or other receptors in the microenvironment of cancer cells. In other words, this active targeting mechanism can inhibit the growth of tumor cells and metastasis, prevent genotypic and phenotypic variations in neovascular endothelial cells, and control drug resistance [107]. Furthermore, some receptors in the tumor microenvironment, such as Vascular endothelial growth factor (VEGF), Vascular cell adhesion protein (VCAM), matrix metalloproteases, and integrin, are targeted in this mechanism [95].

### Cellular Uptake of Therapeutic NPs and the Effect of Liposomal Drugs on Targeted Cells: Actions and Interactions

As it is mentioned earlier, liposomes are able to target tumor cells either passively or actively. After the liposome reaches the cancerous cells and the tumor environment through the targeting mechanism, it can release its therapeutic content and exert its effects by means of various mechanisms. Consequently, lipid composition, the surface charge of the membrane, type of cancer, type of target cells, as well as the presence of specific ligands on the liposome membrane, can influence the cell-liposome interaction [108].

**Fig. 8:**
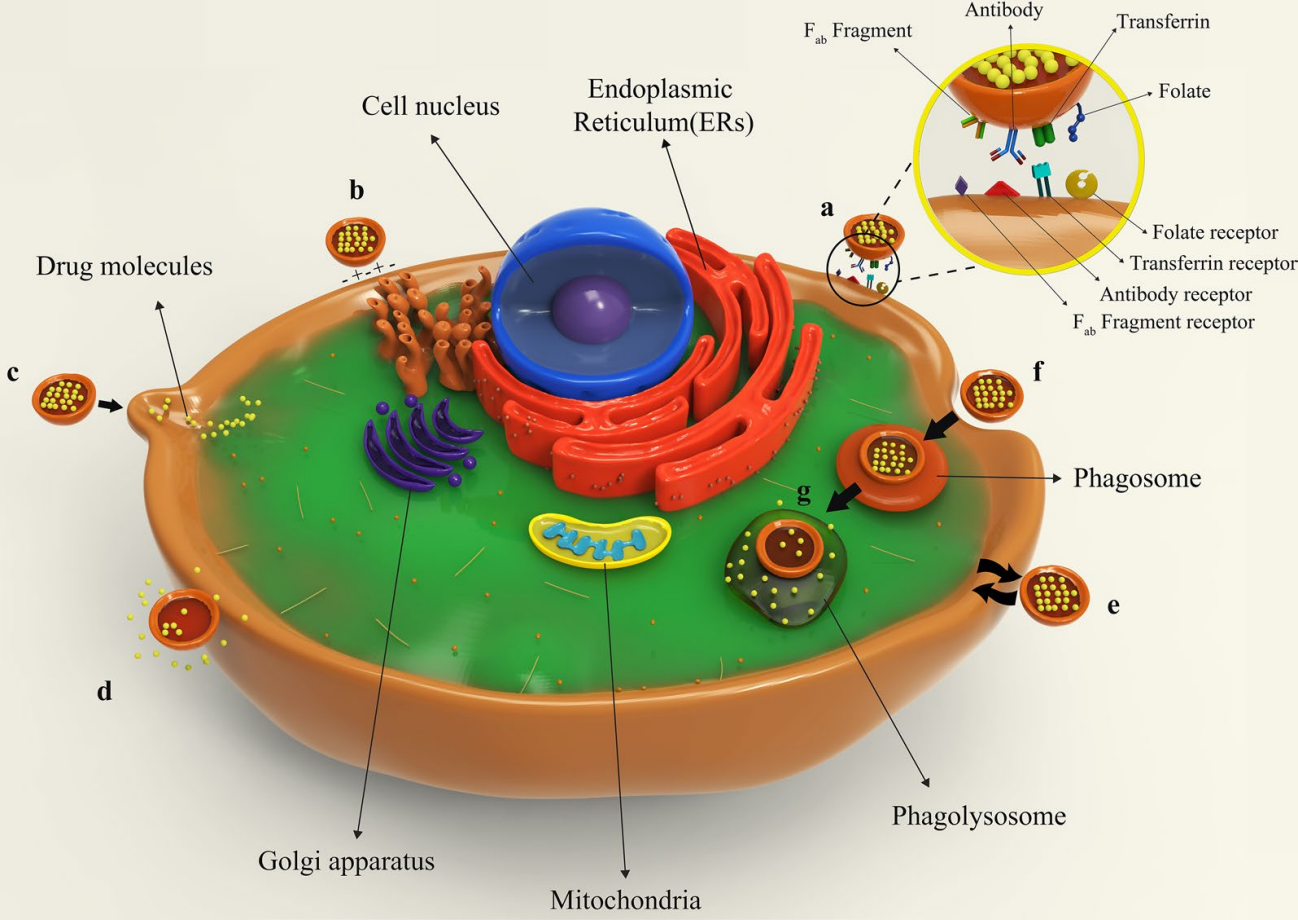
Binding of liposomes to the target cell. **a** Specific attachment via ligand-receptor interaction; **b** non-specific absorption of liposomes through intramolecular-electrostatic forces; **c** the attachment and fusion of liposome to the cell membrane and drug release; **d** liposome arrival to the target cell and drug release without fusion; **e** exchange lipid fragments between the cell membrane and liposome through protein-mediated processes; **f** endocytosis of liposome by target cell; **g** lysosomal digestion of liposome in the cell cytoplasm

Figure 8 illustrates different types of liposomes interactions with target cells. After being injected into the body, drug-containing liposomes travel to different tissues through blood vessels and eventually reach their target cells based on their surface ligands. These liposomes can bind to cellular receptors via these ligands, which is called specific absorption [42]. Albeit, receptor-free liposomes can also adhere to the target cell surface through molecular attractions, electrostatic forces, and molecular interactions called non-specific absorption. Following liposomes binding to the cell, the therapeutic agent is released into the cytoplasm, and its effects may be produced in different ways. The liposomal nanocarriers can be entirely fused to the plasma membrane of the cell and release the drug. Drug compounds are also able to be released from the liposome into the cell and to enter the cell through micropinocytosis or passive diffusion without the occurrence of fusion. Liposomes may directly interact with the cell or exchange lipid fragments with the cell membrane through protein-mediated processes. At the same time, the drug may act on the cell and exert the therapeutic effects of the liposomal drug. However, some liposomes are capable of entering through endocytosis (specific or nonspecific). In particular, liposomes penetrating the cell via this passage can have various destinies. It is possible for them to combine with lysosomes. In such cases, lysosomal enzymes affect the structure of the drug by reducing the pH of the phagolysosome sac. Ultimately, liposomes release the drug by fusing it to the cell membrane or endocytosis, and after that, medications exert their therapeutic effect [42, 62]. All possible ways for the liposome to penetrate the cell and exert its effect are depicted and compared in Fig. 8.

On the other hand, NP-based medications can undergo endocytosis, pinocytosis, or phagocytosis by the target cells. Endocytosis is known as the process in which compounds outside the cell space approach the cell membrane and then enter the cell as a vesicle [109]. Pinocytosis, also recognized as fluid endocytosis, occurs when small molecules or suspensions are introduced into a cell through a vesicle by creating an invagination in the cell membrane. Moreover, pinocytosis vastly occurs in human cells to absorb fat droplets. In an immunological study, Yuriko Tanaka et al. reported that liposome-coupled antigens pinocytosis can be performed by antigen processing cells (APC). This report had proved that liposomes can undergo pinocytosis mechanisms [110]. In phagocytosis, particles larger than 0.5 μm are engulfed by immune cells, it may also occur for liposomes (especially for MLVs and liposomes larger than 500 nm). For example, Jitendra N. Verma et al. confirmed the occurrence of phagocytosis on liposomes by a study on the phagocytosis of liposomes with malarial antigens by macrophages [111].

### Kaposi's Sarcoma, One Instance of Successful Liposomal Drugs Applications

Kaposi's sarcoma is a progressive multifocal anti-proliferative cancer primarily known as endometrial sarcoma. This cancer is more common in HIV patients whose immune system is weakened. Furthermore, it has been commonly seen in skin tissue and may also involve other tissues. Hence, this disorder is generally referred to as skin mucosal sarcoma [112]. To treat this disease, modified long-circulating liposomes can be helpful. In this regard, liposomes passively target tumor cells. Moreover, the effect of EPR and specific binding increases the concentration of the therapeutic drug in cancer tissues 5 to 11 times higher than normal skin [113]. For this purpose, Doxorubicin is used for the treatment of this disease. Correspondingly, entrapment of the doxorubicin into liposomes (which was PEGylated to prolong its half-life) prevents normal tissues from being exposed to the drug. It also reduces drug uptake by these healthy doxorubicin-sensitive tissues such as the heart [114].

**Fig. 9:**
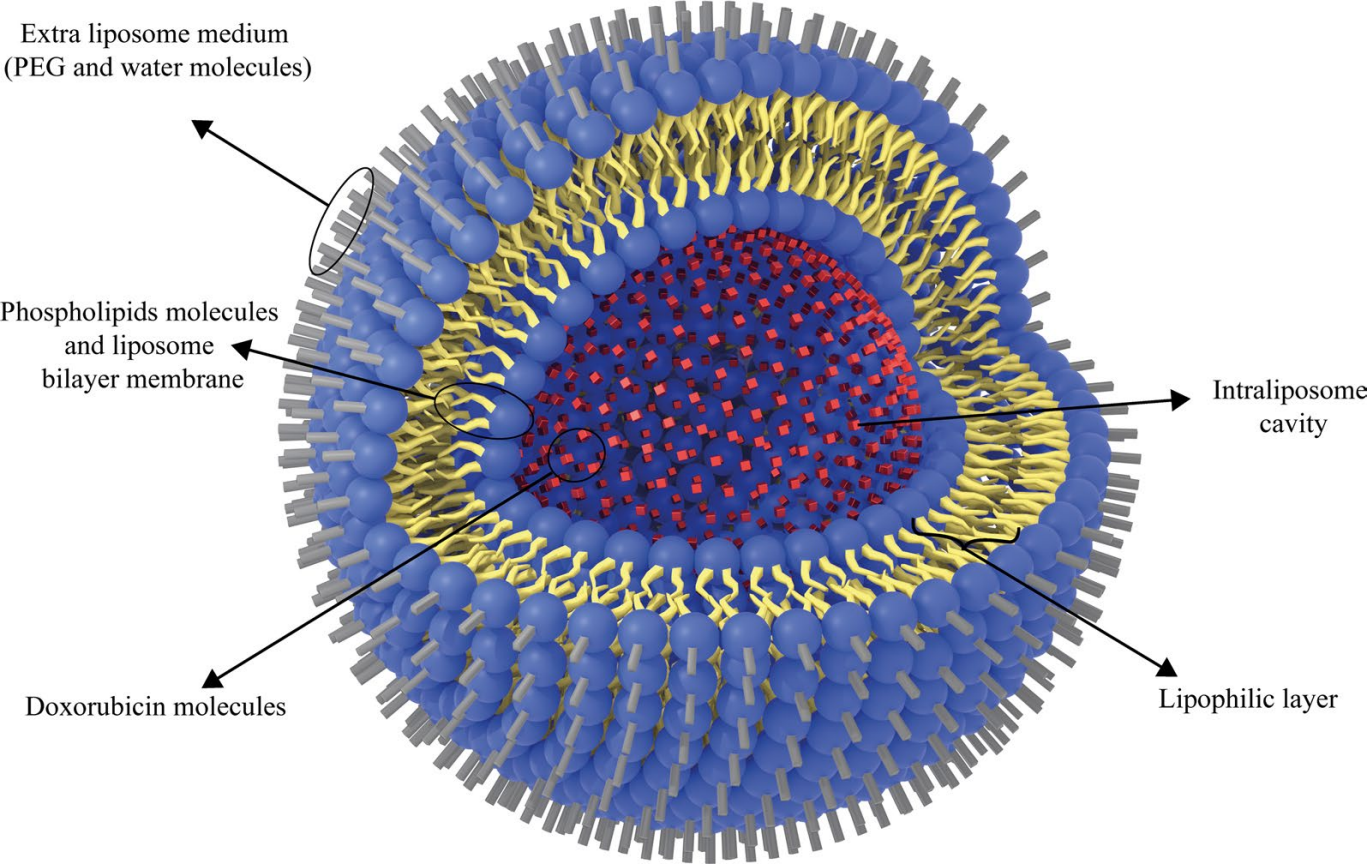
The schematic structure of Doxil drug. Doxorubicin drug molecules are entrapped in the hydrophilic cavity of unilamellar PEGylated liposomes

Additionally, the liposomal form of doxorubicin, Doxil, is a type of anthracycline drug which is approved for clinical administration by US-FDA. It is used to treat AIDS-related Kaposi sarcoma and multiple myeloma [115]. Doxil has better therapeutic efficacy and less toxicity than free doxorubicin, which can be attributed to its ability to target tumors indirectly. It is also passive targeting due to leakage of tumor vessels and the EPR effect [116]. Moreover, the Doxil unilamellar liposomes are < 100 nm in size and have been used to treat various cancer types [42]. Analyses have also proved that free doxorubicin concentration is lower than that of Doxil at the target tissue site [117]. In this regard, Ogawara et al. investigated the effect of Doxil (formed by binding doxorubicin to PEG liposomes) on cancer cells in male mice and showed that PEG liposomal doxorubicin or Doxil1 had been effective on both doxorubicin-resistant and doxorubicin-sensitive C26 cell groups [118]. This can highlight the significance of the exploitation of liposomal NPs. Because they can be consumed to overcome the resistance of cancer cells to common chemotherapy agents at low costs without time-consuming research works to discover new clinical therapeutic compounds [119]. The application of nanoparticles, such as liposomes, to deliver doxorubicin to tumor tissues has been widely investigated. Entrapment of ATP-binding cassette transporter superfamily B member 1 (ABCB1) substrate doxorubicin into liposomes can increase drug uptake and enhance its intracellular distribution within cancer cells, especially ABCB1-expressing cancer cells [120]. The simple structure of Doxil is illustrated in Fig. 9.

Furthermore, liposomal nanomaterials can be exploited for the treatment of infectious diseases. Systemic fungal infection is one of the most challenging conditions that is usually treated with amphotericin B, which is highly toxic to kidney cells. For this purpose, the usage of liposome-entrapped amphotericin B can reduce the toxicity of this drug compared to its free form [48]. Unilamellar liposomes have been used to entrap this agent. It has proven that liposomal amphotericin B is more effective than the free drug form [121]. Based on the formulation, these liposomes also alter the bio-distribution of amphotericin B, such as anticancer drugs, which in turn not only arrange the mechanism of action but also increase the effective dosage concentration at the target tissue [122]. AmbiSome, liposomal form amphotericin B, is approved for public administration too. Other approved liposomal drugs, from anti-fungal medications to cancer therapeutic agents, are summarized in Table 1.

**Table 1:** Liposomal drugs nanoparticles officially approved for various cancers treatment

Product commercial name	Active therapeutic agent	Liposomal nanoparticle platform	Drug category	Indication	Main composition of liposome	Type of infusion	Status	References
AmbiSome	Amphotericin B	PEGylated liposome	Antifungal drug	Aspergillosis Cryptococcosis candidiasis	HSPC, DSPG and cholesterol	I.V	Approved	[42, 123]
DepoDur	Morphine sulphate	Extended-Release Liposome	analgesic drug	General pain reduction	DOPC, DPPG, cholesterol and triolein	Epidural	Approved	[124, 125]
Inflexal	Inactivated hemagglutinin of influenza virus strains A and B	Liposomes	Flu vaccines	Prevention of influenza disease	DSPC and cholesterol	I.M	Approved	[126, 127]
DaunoXome	Daunorubicin citrate	Conventional liposome	anthracyline antitumor antibiotic	Blood cancer HIV-related Kaposi sarcoma	DSPC and cholesterol	I.V	Approved	[36, 42, 127, 128]
Depocyt	Cytarabine	Conventional liposome	Antimetabolites chemotherapy medication	Acute myeloid leukemia (AML), acute lymphocytic leukemia (ALL)	DOPC, DPPG, cholesterol and triolein	Spinal	Approved	[36, 42, 127, 129]
Doxil	Doxorubicin	PEGylated liposome	Anthracyclines chemotherapy drugs	Ovarian and breast cancer, HIV-related Kaposi sarcoma, multiple myeloma	HSPC, cholesterol and DSPE-PEG2000	I.V	Approved	[42, 127, 128]
Epaxal	Inactivated hepatitis A virus (strain RG-SB)	Liposomes	HAV vaccines	Prevention of Hepatitis A virus	DOPC and DOPE	I.M	Approved	[42, 130]
Visudyne	Verteporfin	Conventional liposome	Photosensitizing agents	PDT sensitizer,pathologic myopia, ocular histoplasmosis	EPG and DMPC	I.V	Approved	[42, 130, 131]
Mepact	Muramyl tripeptide phosphatidylethanolamine	Conventional liposome	Immunomodulator antitumor compound	Solid tumors chemotherapy	DOPS and POPC	I.V	Approved	[126, 128, 132]
Nyotran	Nystatin	Conventional liposome	Polyenes antifungal liposomes	Systemic fungal infections	DMPC, DMPG, and cholesterol	I.V	Terminated	[36, 42, 130]
Onivyde or MM-398	Irinotecan	PEGylated liposome	Topoisomerase I inhibitors antineoplastic medications	Post-gemcitabine metastatic Pancreatic cancer	DSPC,MPEG-2000:DSPE	I.V	Approved	[126, 128]
liposomal bortezomib	Bortezomib	PEGylated liposome	Antineoplastic agents	Multiple myeloma	DPPC, MPEG2000-DSP, cholesterol	I.V or S.C	Approved	[38, 133]
Marqibo	Vincristine	PEGylated liposome/ conventional liposome	Anti-malignant agents	Philadelphia chromosome-negative acute lymphoblastic leukemia	Sphingomyelin, cholesterol	I.V	Approved	[38, 134, 135]

Although the application of liposomal NPs to treat cancer has been touted as a viable solution for drug delivery and affecting tumor cells, drug delivery to cancerous tissues in the central nervous system (CNS) has remained a significant challenge. In addition, drug delivery to central nervous system cells faces many turbulences owing to a blood–brain barrier (BBB). However, this problem can be partially solved by developing new methods and using lipid-based compounds [136].

### Liposomal Nanoparticles in the Investigational Phase for Therapeutic Purposes

#### Liposomal siRNA

RNA is a type of genetic molecule with a variety of functions, including translation and transcription processes. The discovery of small-interfering RNA (siRNA) is a significant advance in biology in the last decade [137]. Synthetic siRNAs can be utilized to target oncogenes and their mRNAs. Furthermore, siRNAs can be applied for targeting genes contributing to the carcinogenesis, proliferation, and metastasis of tumor cells or their resistance to standard chemotherapies and radiation [138]. Therefore, it has been considered a modern method for cancer therapy. On the other hand, the nanoparticles used to deliver siRNA must possess properties such as biodegradability, great bio-distribution, low toxicity, etc. All of these features can be offered by liposomes making this popular drug delivery system a promising candidate for this purpose [28]. siRNAs bound to neutral lipid-based NPs are well isolated from these liposomes. They also influence ephrin type-A receptor 2 (EphA2), focal adhesion kinase (FAK), neuropilin-2, Interleukin 8 (IL-8), and TROJAN Mobile Remote Receiving System/erythroblast transformation-specific (TMRRS/ERG), Elongation factor 2 kinase (EF2K) or Bcl-2 pathways. Following the occurrence of this mechanism, a suitable antitumor effect has been observed against ovarian, colon, and breast cancer cells, etc. [139, 140]. Numerous studies have been conducted on siRNA delivery by liposomes, and in most of them, the cationic lipid Dioleoyl-3-trimethylammonium propane (DOTAP) has been widely expended in the structure of liposomes. Due to DOTAP high positive charge, this cationic lipid can be toxic to cells. It can stimulate cellular hemolysis and reduce ultimate biocompatibility as well. This has challenged the application of this lipid in the composition of liposomes applied for siRNA delivery [141].

#### Liposomal Curcumin Nanoparticles

**Fig. 10:**
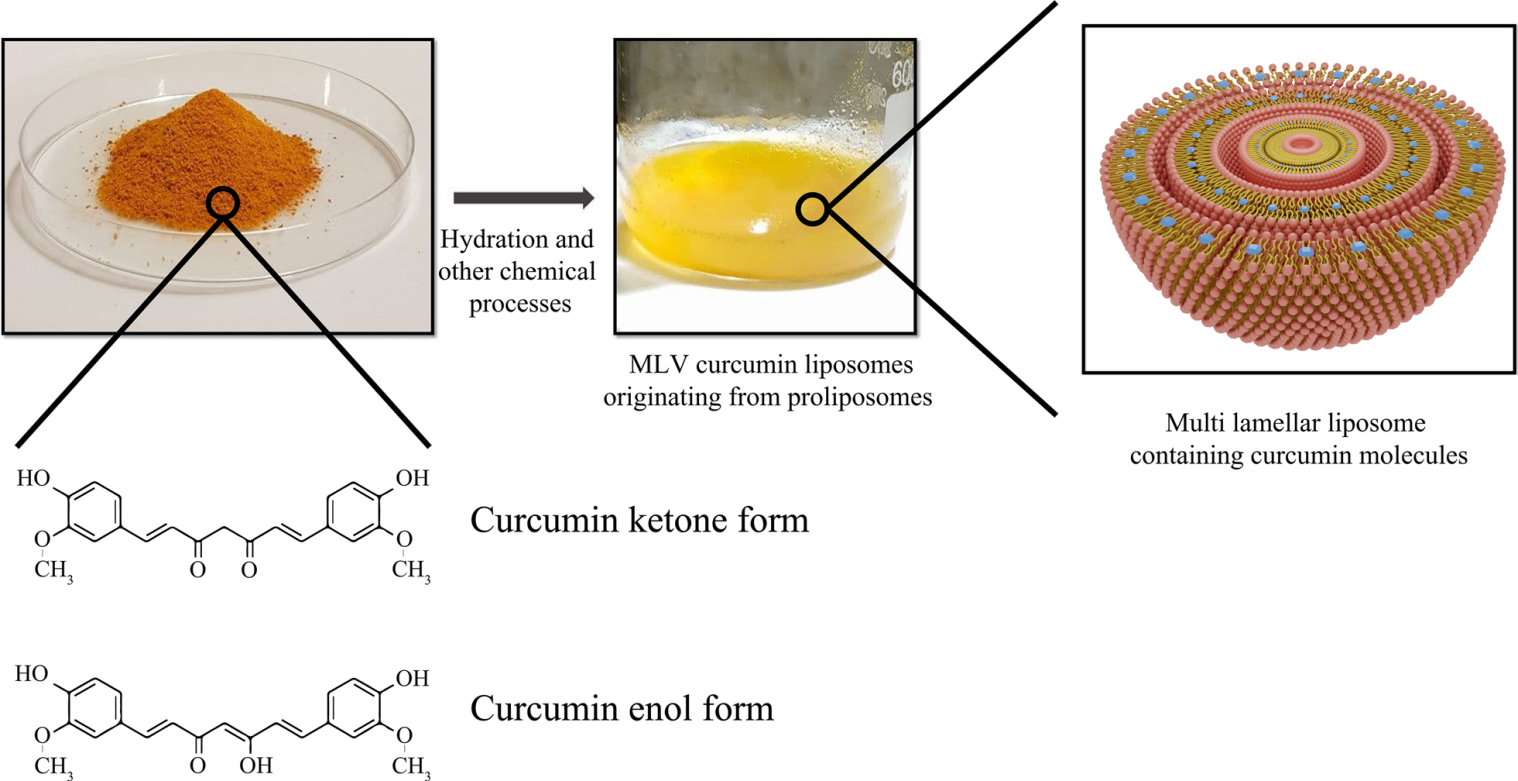
An overview of curcumin powder and liposomal curcumin synthesis. Chemical reactions performed for liposomal curcumin production and curcumin molecule structure in various forms are simply demonstrated

Curcumin-conjugated liposomes are another instance of liposomal nanoparticle usage. Curcumin is a natural polyphenolic and hydrophilic compound that is abundant in the *Curcuma longa* plant and can be mainly prepared from turmeric extraction. Nowadays, the anticancer effect of curcumin has been well indicated against many tumor cells, such as breast cancer, liver carcinoma, and prostate cancer, etc. [142]. The primary mechanism of action of curcumin against cancer cells is to interfere with the translation of proteins such as Bcl-xl and regulate apoptosis by influencing their process, controlling the release of reactive oxygen species (ROS) and cytochrome, regulating molecular factors such as cyclin affecting the cell cycle. On the other hand, curcumin can damage the nuclear and mitochondrial DNA structure of liver cancer cells, thereby disrupting their function [143]. In comparison with free curcumin, the application of liposomal curcumin improves pharmacokinetics and pharmacodynamics while reducing the dosage required to target tumors. Matheus Andrade Chave et al. explored curcumin-containing liposomes by inserting curcumin molecules into the MLV liposome [144]. The synthesis of liposomal curcumin and curcumin structure are described in Fig. 10.

In addition, liposomes prepared for therapeutic research applications can be synthesized by employing various methods. For example, Qiao Wang et al. exploited the ultrasonication and lipid film-hydration method to synthesize daidzein long-circulating liposomes (DLCL) [145]. Xiaoyuan Ding et al. also used the film hydration method for the synthesis of aptamer and Au-NPs (Apt-Au)-modified Morin pH-sensitive liposome. Their outcomes showed high biocompatibility and insignificant toxicity of these liposomal structures and highlighted these liposomes as a viable option for selective targeting of tumors [146].

#### Other Liposomal NPs in the Investigational Phase

Several liposomal drugs have been synthesized and utilized in various medications at the investigational phase. For instance, CPX-1 was produced by entrapping the antitumor agents, Irinotecan and floxuridine (1: 1 molar ratio) in liposomes, and was designed to treat advanced colorectal cancer. This therapeutic nanoparticle is in phase II research status [128]. Lipovaxin-MM is another momentous liposomal nanoparticle in phase I research prepared by placing melanoma antigens in liposomes and mainly administrated for immunotherapy of malignant melanoma. This agent is also under investigation [128].

## Conclusion

As spherical structures in liquids, liposomes can be applied as a promising option for cancer therapy and drug delivery, as well as imaging, and disease management. By reviewing liposomes pros and cons, scientists will be able to improve them in future research works.

Some opportunities and challenges in liposomes utilization are described in the following. One of the convenient features of liposomes is their morphological similarity to cells (presence of phospholipids), as well as increasing the effectiveness of the drugs. As a negative point, liposomal phospholipids may sometimes undergo hydrolysis or oxidation reactions which may be problematic. Other pros of liposomes include increased stability of the encapsulated drug in it, reduced contact of sensitive tissues with therapeutic molecules, decreased drug toxicity, improved pharmacokinetic and pharmacodynamics properties, the ability to regulate the rate of drug release, and the potential of their structure to accept the desired chemical modification. In contrast to these opportunities, there are some challenges such as leakage or unintended entrapment of drugs, low liposome bioactivity, decreased-solubility, rapid clearance of conventional liposomes from the blood by the reticuloendothelial system (RES), and problems caused by continuous intravenous administration or local injection.

Besides examining the advantages and disadvantages of liposomes, we should take their proper targeting mechanism of action into account. Passive targeting is considered a beneficial mechanism due to the abundant clinical evidence and experience. It also increases the circulation time of liposomal drugs. The problem of this mechanism lies in its non-specific drug delivery and its physiological barriers. In contrast, beneficial features of active targeting include increased specificity in drug delivery, the possibility of overcoming chemotherapy-resistant tumor cells, and reduced off-target effects. However, the difficulty in identifying accurate binding sites on cancer cells and the lack of adequate evidence of its former utilization have led to some ups and downs in its application.

Liposomes are reasonable candidates for elevating the effectiveness of current anticancer agents and preventing the incidence of drug resistance. Future research in this area should be focused on further investigation into the properties of liposomal structures. To probe about drug entrapment in therapeutic nanoparticles, including liposomes, much more detailed examinations will be required.

## Abbreviations

WHO: World Health Organization
VNPs: Viral nanoparticles
NPs: Nanoparticles
US FDA: United States Food and Drug Administration
DDSs: Drug delivery systems
PC: Phosphatidylcholine
SM: Sphingomyelin
PS: Phosphatidylserine
PE: Phosphatidylethanolamine
ULVs: Unilamellar vesicles
OLVs: Oligo lamellar vesicles
MLVs: Multilamellar vesicles
SUVs: Small unilamellar vesicles
RBF: Round-bottom flask
LNs: Liposomal nanoparticles
MPS: Mononuclear phagocytosis system
PEG: Polyethylene glycol
PEOZ: Poly [2-ethyl 2-oxazoline]
PETOXylated: Poly [2-ethyl-2-oxazolylated
PMOZ: Poly [2-methyloxazoline]
HDL: High-density lipoprotein
LDL: Low-density lipoprotein
SCLC: Small cell lung cancer
GRPR: Gastrin-releasing peptide receptor
Anti-EpCAM: Anti-epithelial cell adhesion molecule
IUPAC: International Union of Pure and Applied Chemistry
I.V.: Intravenous
S.C.: Subcutaneous
I.D.: Intradermal
I.P.: Intraperitoneal
I.M.: Intramuscular
EPR: Enhanced permeability and retention
fR: Folate receptor
TfR: Transferrin receptor
EGFR: Epidermal growth factor receptor
VEGF: Vascular endothelial growth factor
VCAM: Vascular cell adhesion protein
APC: Antigen-presenting cells
ABCB1: ATP-binding cassette transporter superfamily B member 1
HSPC: Hydro soy phosphatidylcholine
DSPG: 1,2-Distearoyl-sn-glycero-3-PG
DOPC: Dioleoylphosphatidylcholine
DPPG: 1,2-Dipalmitoyl-sn-glycero-3-phosphoglycerol
DSPC: 1,2-Distearoyl-sn-glycero-3-phosphocholine
AML: Acute myeloid leukemia
ALL: Acute lymphocytic leukemia
DSPE: 1,2-Distearoyl-sn-glycero-3-phosphoethanolamine
DOPE: Dioleoylphosphatidylethanolamine;
EPG: Esterified propoxylated glycerols;
DMPC: 1,2-Dimyristoyl-sn-glycero-3-phosphocholine
DOPS: 1,2-Dioleoyl-sn-glycero-3-phospho-L-serine
POPC: 1-Palmitoyl-2-oleoyl-sn-glycero-3-phosphocholine
DMPG: 1,2-Dimyristoyl-sn-glycero-3-phosphoglycerol
MPEG: Methoxypolyethylene glycols.
CNS: Central nervous system
BBB: Blood–brain barrier
siRNA: Small-interfering RNA
EphA2: Ephrin type-A receptor 2
FAK: Focal adhesion kinase
IL-8: Interleukin-8
TMRRS/ERG: TMRRS/ERG TROJAN Mobile Remote Receiving System/erythroblast transformation-specific
EF2K: Elongation factor 2 kinase
DOTAP: Dioleoyl-3-trimethylammonium propane
ROS: Reactive oxygen species
DLDC: Daidzein long-circulating liposomes
RES: Reticuloendothelial system
